# Supplementary material for: Enhanced CD95 and interleukin 18 signalling accompany T cell receptor Vβ21.3+ activation in multi-inflammatory syndrome in children
Source: Nat Commun. 2024 May 18;15:4227. doi: 10.1038/s41467-024-48699-y (PMC11102542; doi:10.1038/s41467-024-48699-y)
Supplement: Supplementary file 1 — Supplementary Information [file 41467_2024_48699_MOESM1_ESM.pdf]

Supplementary information

Enhanced CD95 and IL-18 Signaling Accompany TCR Vβ21.3+ T Cell Activation in MIS-C

Zhenguang Zhang[1], Iain R.L. Kean[1], Lisa M. Dratva[2], John A. Clark[1], Eleni Syrimi[3], Naeem Khan[3], Esther Daubney[4], Deborah White[4], Lauran O'Neill[5], Catherine Chisholm[5], Caroline Payne[5], Sarah Benkenstein[5], Klaudia Kupiec[5], Rachel Galassini[6], Victoria Wright[6], Helen Winmill[7], Ceri Robbins[7], Katherine Brown[5], Padmanabhan Ramnarayan[6], Barnaby Scholefield[7,8], Mark Peters[5,9], Nigel Klein[5,9], Hugh Montgomery[10], Kerstin B. Meyer[2], Sarah A. Teichmann[2,11], Clare Bryant[12]*, Graham Taylor[3]*, Nazima Pathan[1, 4]*.

Corresponding authors: Dr Nazima Pathan: np409@cam.ac.uk, Department of Paediatrics, School of clinical medicine, University of Cambridge, UK; Dr Graham Taylor: g.s.taylor@bham.ac.uk, Institute of Immunology and Immunotherapy, University of Birmingham, UK and Professor Clare Bryant: ceb27@cam.ac.uk, Department of Medicine, University of Cambridge, UK.

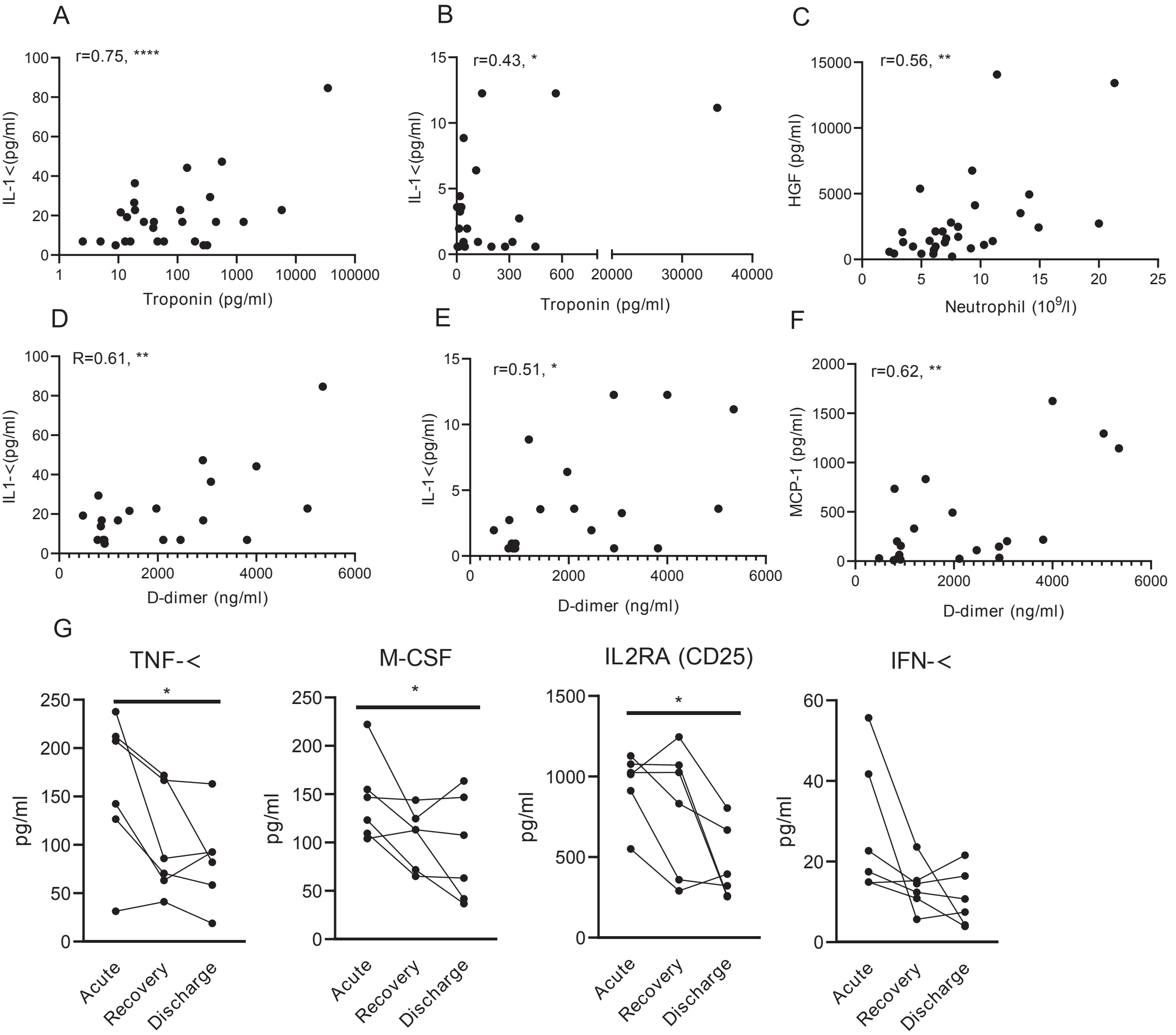

Supplementary Figure 1 Correlation between cytokines and clinical test markers, temporal changes in cytokines in MIS-C. Related to Figure 2. (A) Correlation between IL1-α and troponin. (B) Correlation between IL-1β and troponin. (C) Correlation between HGF and neutrophil count. (D) Correlation between IL-1α and D-dimer. (E) Correlation between IL1β and D-dimer. (F) Correlation between MCP-1 and D-dimer. Pearson correlation test was used: * = p<0.05, ** = p<0.01. (G) Plasma levels of TNF-α, M-CSF, IL2RA, and IFN-γ for six MIS-C patients at acute, recovery (2-4 days after PICU admission) and follow-up (approximately one month after discharge) time points. Source data are provided as a Source Data file.

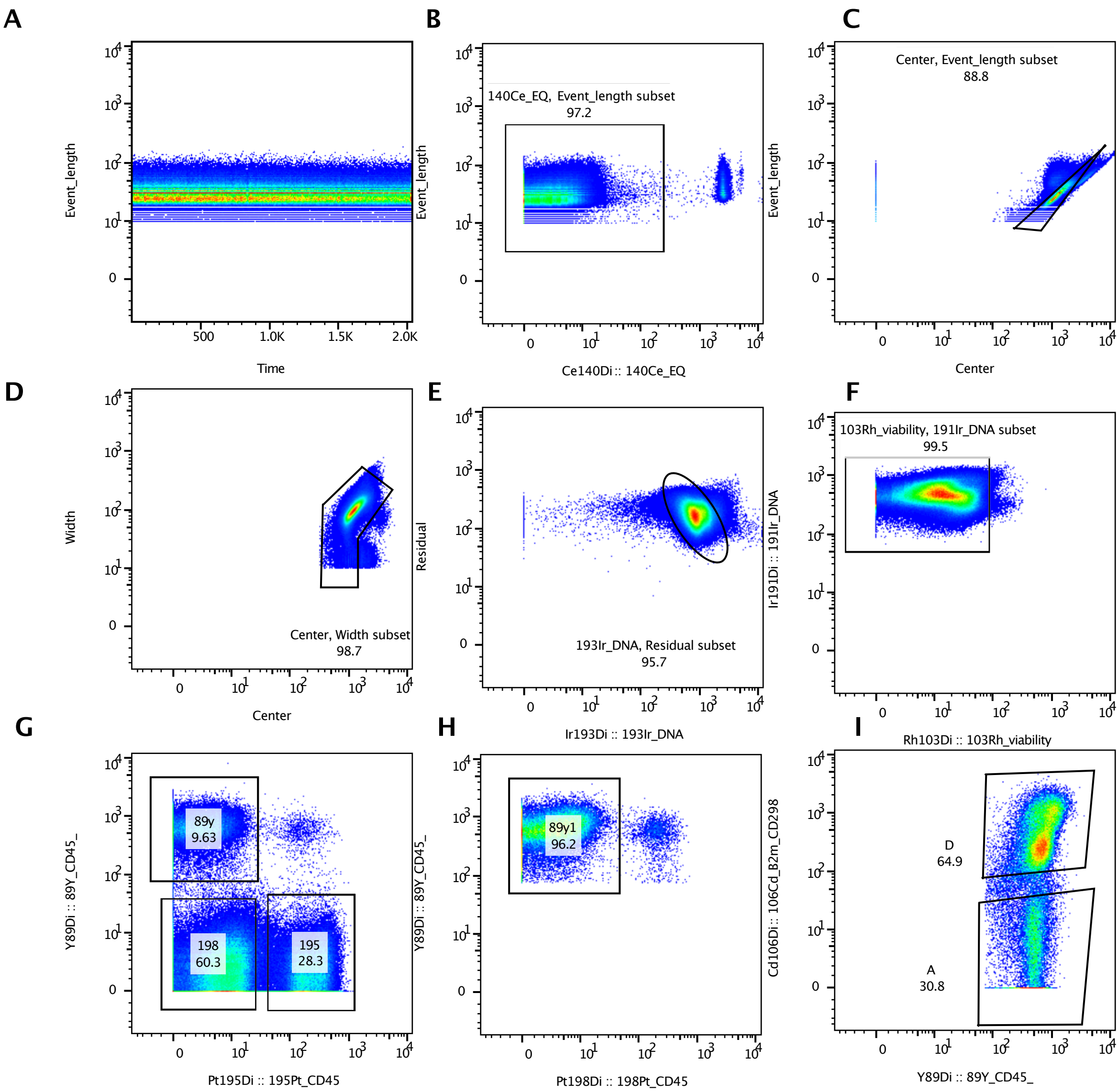

Supplementary Figure 2 Cleaning and unmixing of CyToF data. Related to Figure 3. (A) Plotting of events over time. (B) Gating to remove beads. (C)–(F) Gating of single live cells using event length, width, and residual parameters followed by iridium DNA dye and rhodium live/dead dye. (G)-(I) Example of unmixing of pooled samples, labelled with CD45-89Y, CD45-195Pt, CD45-98Pt and B2M/ CD298-106Cd.

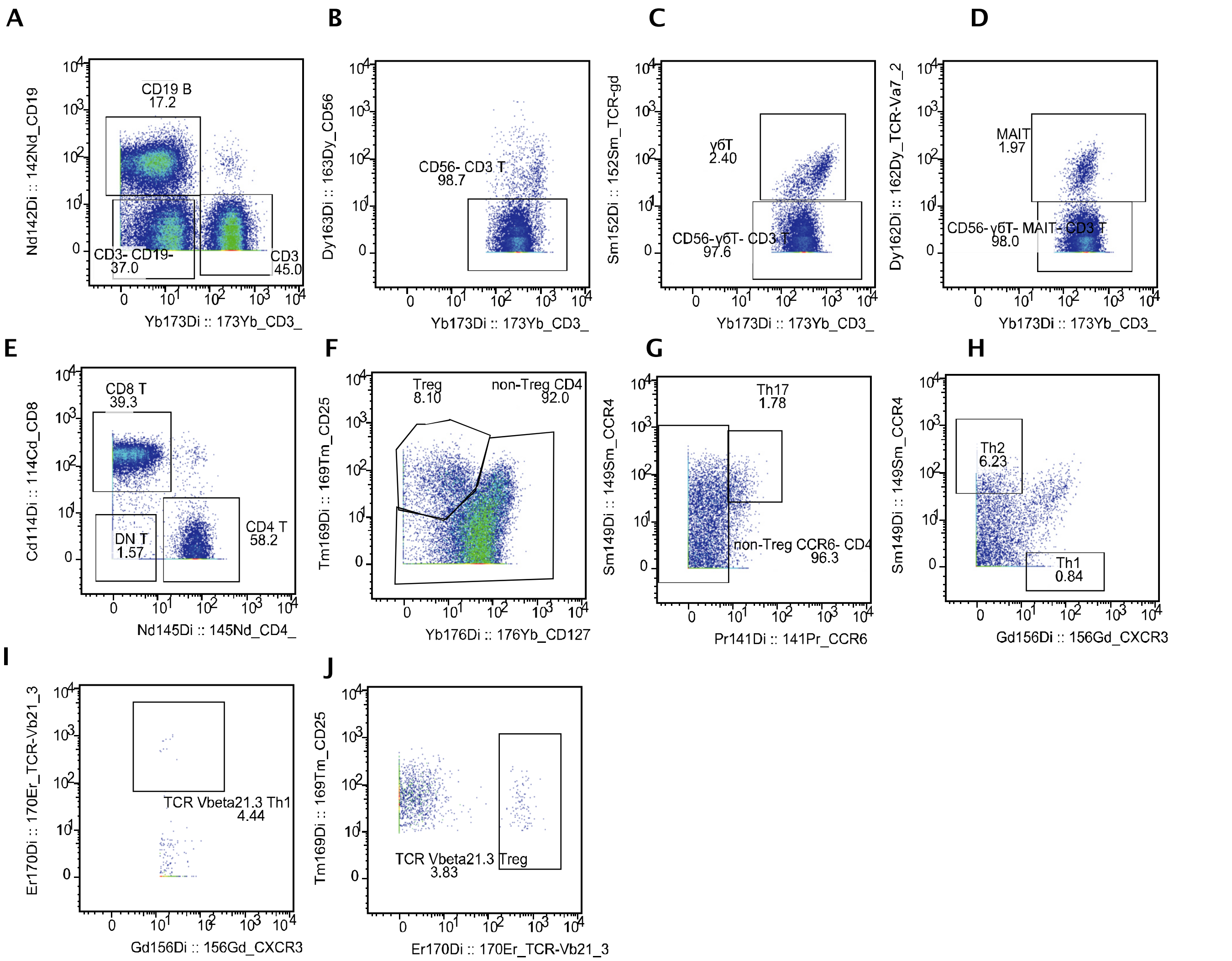

Supplementary Figure 3 Manual gating of different T cell types using Flowjo software. Related to Figure 3. (A) Gating using CD3 and CD19 parameters to gate CD3+ T cells. (B) Excluding NK cells by gating on CD56-negative events. (C) Excluding γδ T-cells by gating on TCRγδ-negative events. (D) Excluding MAIT cells by gating on TCR-Vα7.2 negative events. (E) Gating of CD4+ and CD8+ T cells from clean CD3+ T cells selected as shown in panels A-D. (F) Gating of CD25high CD127low Treg cells from CD4+ T cells. (G) Gating of CCR4+ CCR6+ Th17 cells from non-Treg cells. (H) Gating of CXCR3+ Th1 cells and CCR4+ Th2 cells from negative population in (F). (I and J) Gating of TCR-Vβ 21.3+ events from Th1 and Treg, respectively.

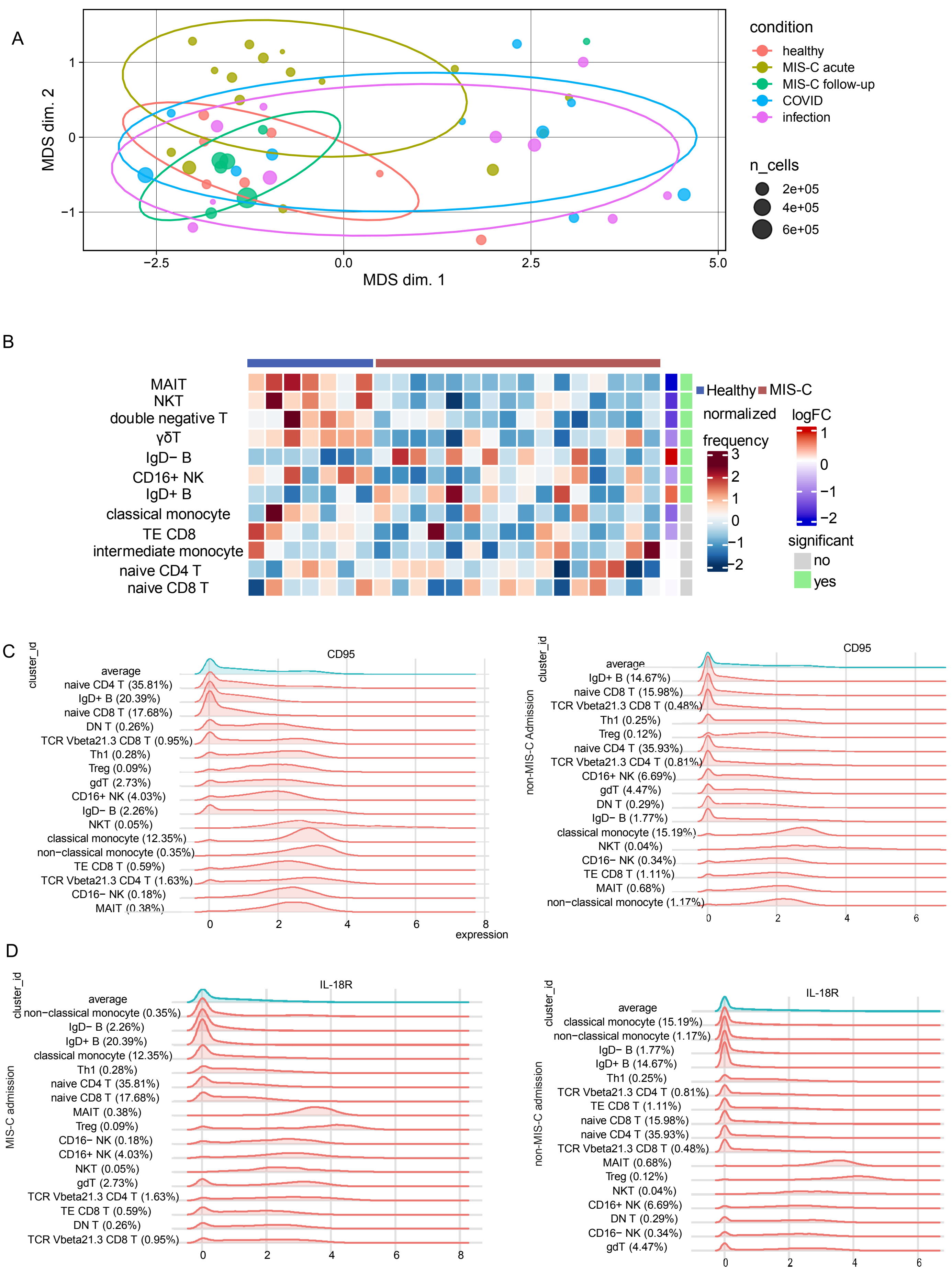

Supplementary Figure 4 Analysis of T cell panel CyToF data by CATALYST package. Related to Figure 3. (A) MDS plotting of samples by cell type markers. (B) Heatmap of cell population abundance comparing MIS-C acute with healthy volunteer samples. (C and D) Expression levels of CD95 (C) or IL-18R (D) in different populations of immune cells in PBMCs from acute MIS-C patients or all other samples; cell populations on the Y axis were ordered by Euclidean distance of CD95 and CD-18R expression in (C) and (D), respectively.

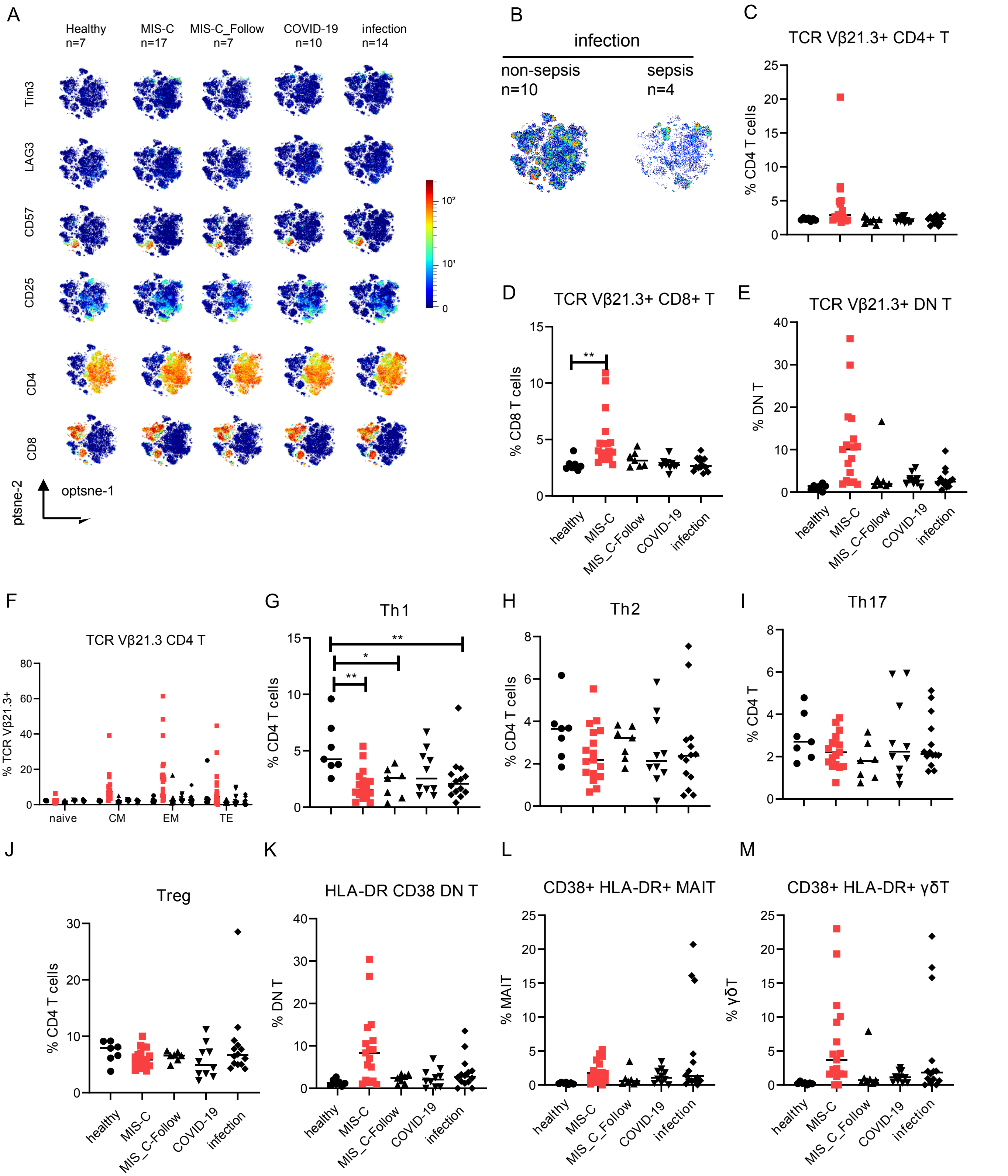

Supplementary Figure 5 Additional data from CyToF analysis using the T-cell panel of antibodies. Related to Figure 3. (A) opt-tSNE projections showing levels of CD4, CD8, TIM3, LAG3, CD25, CD57 markers in the non-naïve T cell populations from the indicated groups of subjects. (B) op-tSNE plotting of non-naïve T cell density in the infectious disease group split by sepsis and non-sepsis conditions. (C) Frequency of TCR Vβ 21.3+ cells in CD4 T cells. (D) Frequency of TCR Vβ 21.3+ cells in CD8 T cells. (E) Frequency of TCR Vβ 21.3+ cells in DN T cells. (F) Percentage of TCR Vβ21.3+ T cells in different subsets of CD4 T cells (naïve, central memory, effector memory, terminal effector). (G)-(J) Frequency of Th1, Th2, Th17 and Treg in CD4 T cells. (K) Frequency of CD38+ HLA-DR+ activated T cells in DN T cells. (L) Frequency of CD38+ HLA-DR+ activated T cells in MAIT cells. (M) Frequency of CD38+ HLA-DR+ activated T cells in γδT cells. Ordinary one-way ANOVA was conducted, with Dunnett's multiple comparisons test comparing other groups with MIS-C group: * = p<0.05, ** = p<0.01, *** = p<0.001, **** = p<0.0001. Source data are provided as a Source Data file.

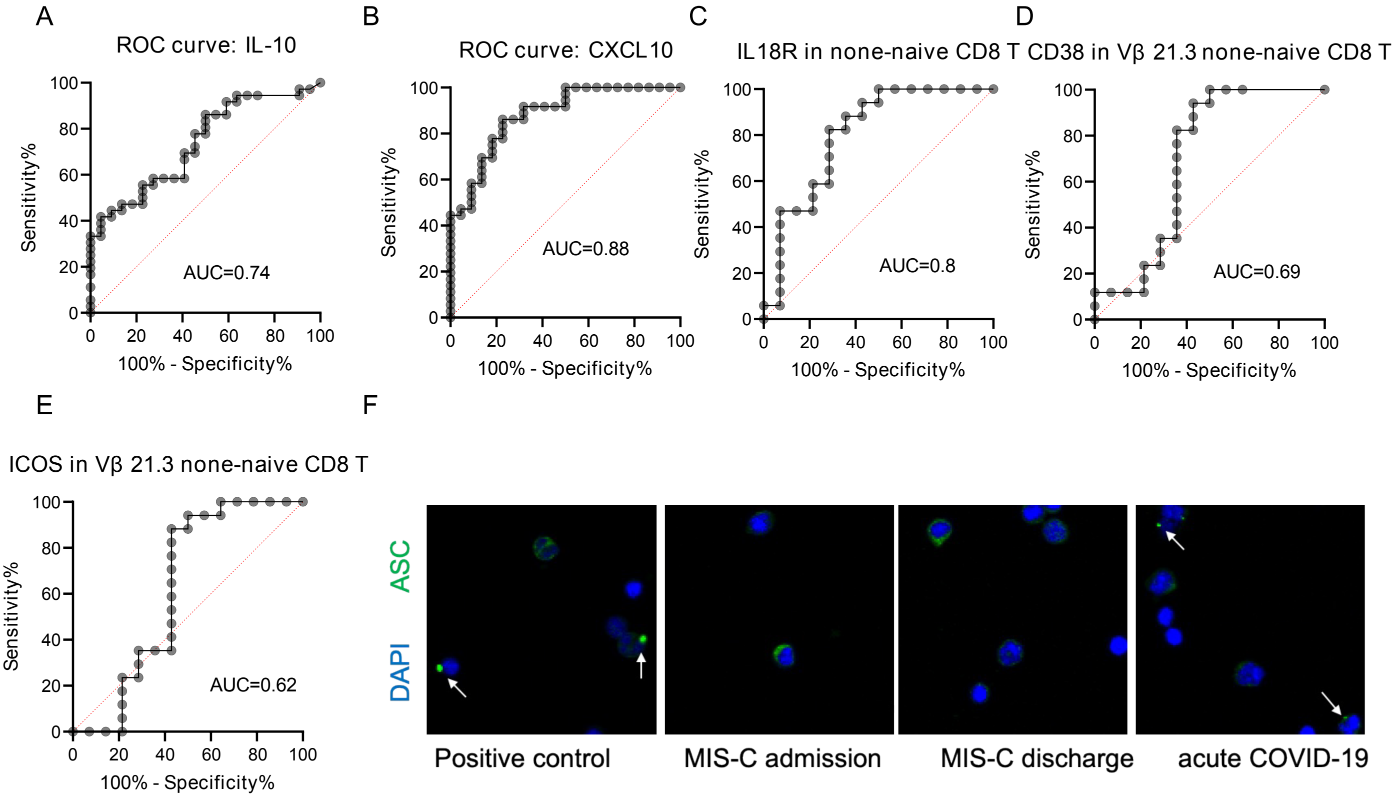

Supplementary Figure 6 ROC analysis using additional markers and ASC staining. Related to Figure 4 and 5. (A) ROC analysis of IL-10 comparing MIS-C samples with respiratory inflammation group as control. (B) ROC analysis of IL-10 comparing MIS-C samples with respiratory inflammation group as control. (C)-(E) ROC analysis of IL-18R, CD38 and ICOS in non-naïve CD8 T cells comparing MIS-C samples with infection samples as control. (F) Representative images from samples where ASC was stained in whole blood fixed with cytodelics kits, including positive control (healthy subject blood samples stimulated with LPS plus ATP to induce NLRP3 activation), MIS-C sample (n=3), MIS-C discharge sample (n=2) and acute COVID-19 sample(n=1). ASC specks can be seen in the positive samples (pointed by white arrow), as well as acute COVID-19 samples, but not MIS-C or MIS-C follow-up samples. Blue staining: DAPI, green staining: ASC. Source data are provided as a Source Data file.

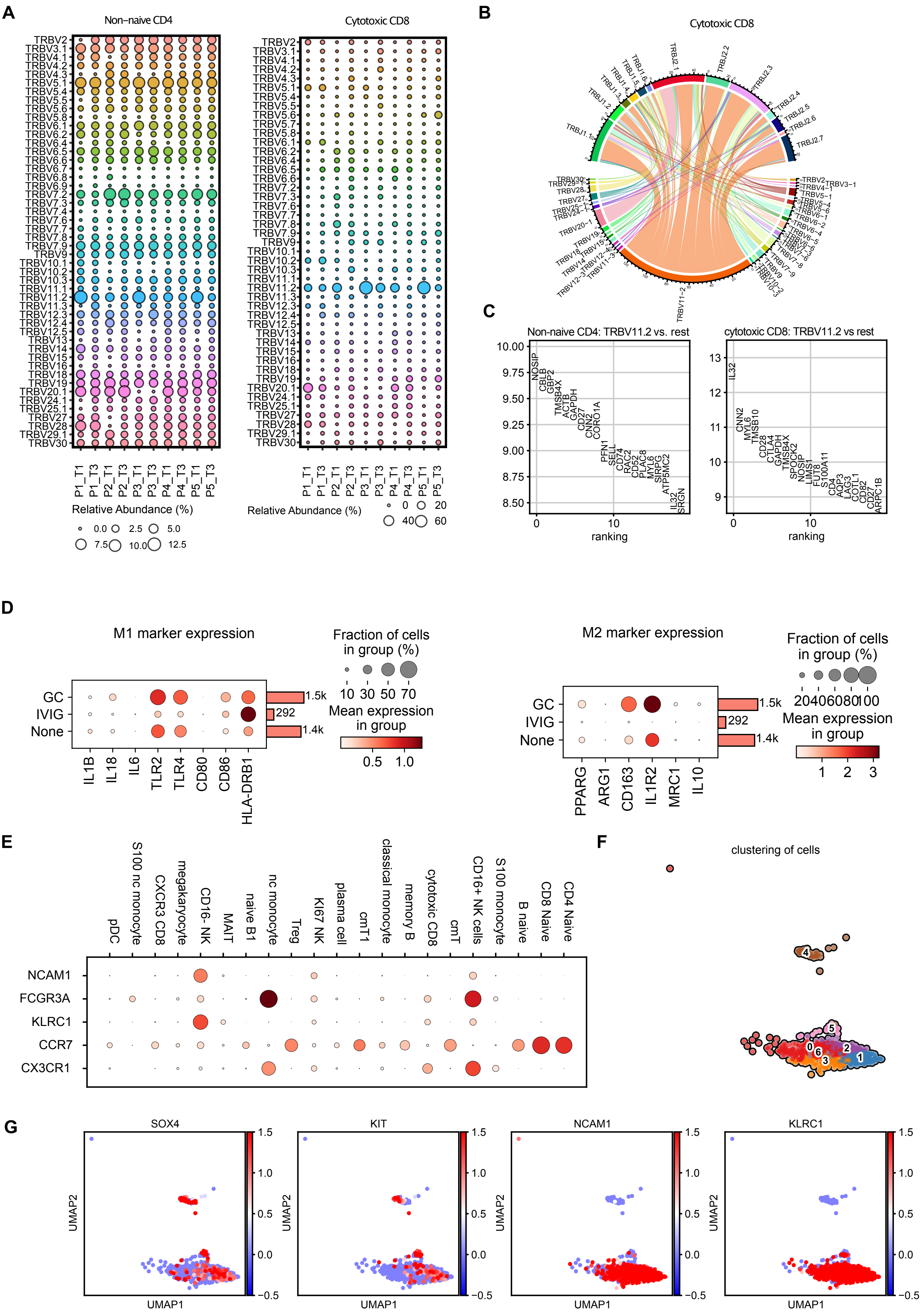

A
Non−naive CD4
Cytotoxic CD8
TRBV2
TRBV3.1
TRBV4.1
TRBV4.2
TRBV4.3
TRBV5.1
TRBV5.4
TRBV5.5
TRBV5.6
TRBV5.8
TRBV6.1
TRBV6.2
TRBV6.4
TRBV6.5
TRBV6.6
TRBV6.7
TRBV6.8
TRBV6.9
TRBV7.2
TRBV7.3
TRBV7.4
TRBV7.6
TRBV7.7
TRBV7.8
TRBV7.9
TRBV9
TRBV10.1
TRBV10.2
TRBV10.3
TRBV11.1
TRBV11.2
TRBV11.3
TRBV12.3
TRBV12.4
TRBV12.5
TRBV13
TRBV14
TRBV15
TRBV16
TRBV18
TRBV19
TRBV20.1
TRBV24.1
TRBV25.1
TRBV27
TRBV28
TRBV29.1
TRBV30
P1_T1
P1_T3
P2_T1
P2_T3
P3_T1
P3_T3
P4_T1
P4_T3
P5_T1
P5_T3
Relative Abundance (%)
0.0
2.5
5.0
7.5
10.0
12.5
0
20
40
60
B
Cytotoxic CD8
C
Non-naive CD4: TRBV11.2 vs. rest
cytotoxic CD8: TRBV11.2 vs rest
NOSIP
CBLB
GBP2
TMSB4X
ACTB
GAPDH
CD27
CNN2
CORO1A
PFN1
SELL
CD74
RAC2
CD52
PLAC8
MYL6
SIRPG
ATP5MC2
IL32
SRGN
CNN2
MYL6
TMSB10
CD28
CTLA4
GAPDH
TMSB4X
SPOCK2
NOSIP
LIMS1
FUT8
S100A11
CD4
AQP3
LAG3
COTL1
CD82
CD27
ARPC1B
ranking
D
M1 marker expression
Fraction of cells in group (%)
10 30 50 70
Mean expression in group
0.5 1.0
GC
IVIG
None
1.5k
292
1.4k
IL1B
IL18
IL6
TLR2
TLR4
CD80
CD86
HLA-DRB1
M2 marker expression
20406080100
1 2 3
PPARG
ARG1
CD163
IL1R2
MRC1
IL10
E
pDC
S100 nc monocyte
CXCR3 CD8
megakaryocyte
CD16- NK
MAIT
naive B1
nc monocyte
Treg
KI67 NK
plasma cell
cmT1
classical monocyte
memory B
cytotoxic CD8
cmT
CD16+ NK cells
S100 monocyte
B naive
CD8 Naive
CD4 Naive
NCAM1
FCGR3A
KLRC1
CCR7
CX3CR1
F
clustering of cells
G
SOX4
KIT
NCAM1
KLRC1
UMAP1
UMAP2

Supplementary Figure 7 Additional scRNA-seq analysis. Related to Figure 6. (A) Plot showing the abundance of all TCR Vβ subtypes in non-naïve CD4 and cytotoxic CD8 T cells from five MIS-C patients, showing samples collected during the acute phase of disease and at their follow-up visit approximately one month after hospital discharge. Patient code is shown on the X axis, with suffix T1 or T3 indicating acute or follow up stage respectively. (B) Vβ and j chain pairing in acute MIS-C cytotoxic CD8 T cells. (C) Top 20 genes differentially expressed between TCR Vβ21.3+ and TCR Vβ21.3– populations in central memory CD4 T (left) and cytotoxic CD8 T (right) cells. (D) Expression of M1 and M2 markers in acute MIS-C monocytes. There are 2 samples without intravenous immunoglobin (IVIG) or glucocorticoid pre-treatment, 1 pre-treated with IVIG and 2 with glucocorticoid. (E) Expression levels of NCAM1 (CD56), FCGR3A (CD16), KLRD1, CCR7, CX3CR1 in all clusters were shown in the violin plots. (F) CD16- NK cells were re-clustered and cluster 0, 1, 2, 3, 6 were selected as true CD16- NK cells for further comparison between admission and follow-up group. IL18 showed a significant change again. (G) Markers of SOX4, KIT, NCAM1, KLRC1 in the CD16- NK cells. Note high SOX4 and KIT expressing cells in cluster 4 and 5 of (F), markers of hematopoietic stem cell.

Metal	antibodies	Source	Identifier	format	Dilution (antibody volume (ul) for 100ul staining volume)
089Y	anti-human CD45 (Clone: HI30)	Biolegend	304002	self labelled	2
106 Cd	anti-human β2-microglobulin (Clone: 2M2)	Biolegend	316302	self-labelled	0.5
110 Cd	Anti-CD14 (Clone: RMO52)	Beckman Coulter	IM0643	self labelled	0.5
111 Cd	Anti-CD38 (Clone: HIT2)	Biolegend	303502	self-labelled	0.75
114 Cd	Anti-CD8 (Clone: RPA-T8)	Biolegend	301002	self labelled	0.4
115 In	Anti-CD57 (Clone: HCD57)	Biolegend	359602	self-labelled	0.5
141 Pr	CCR6 (Clone: G034E3)	Biolegend	353402	self-labelled	0.7
142 Nd	Anti-CD19 (Clone: HIB19)	Fluidigm	3142016 D	commercially conjugated	0.5
143 Nd	Anti-Human CD5 (Clone: UCHT2)	Fluidigm	3143007 B	commercially conjugated	0.5
145 Nd	Anti-Human CD4 (Clone: RPA-T4)	Fluidigm	3145001 B	commercially conjugated	0.5
146 Nd	anti-TIGIT (Clone: A15153G)	Biolgend	372702	self-labelled	1
147 Sm	anti-human CD366 (Tim-3) (Clone: F38-2E2)	Biolegend	345002	self-labelled	1
148 Nd	Anti-CD95 (Clone: DX2, purified)	Biolegend	305602	self-labelled	1
149 Sm	CCR4 (Clone: L291H4)	Biolegend	359402	self-labelled	0.5
150 Nd	Anti-Human CD223/LAG-3 (Clone: 11C3C65)	Fluidigm	3150030 B	commercially conjugated	0.5
151 Eu	TCRVa24 (Clone: 6B11)	Biolegend	342902	self-labelled	1
152 Sm	Anti-Human TCRgd (Clone: 11F2)	Fluidigm	3152008 B	commercially conjugated	1
153 Eu	anti-CX3CR1 (Clone: 2A91)	Biolegend	341602	self-labelled	1
154 Sm	anti-human IgD (Clone: IA6-2)	Biolegend	348202	self-labelled	0.25
155 Gd	anti-CD45RA (Clone: HI100)	Fluidigm	3155011 B	commercially conjugated	0.5
156 Gd	anti-CXCR3 (Clone: G025H7)	Fluidigm	3156004 B	commercially conjugated	0.7
159 Tb	CD28 (Clone:CD28.2)	Biolegend	302902	self-labelled	0.75
160 Gd	Anti-Human CD39 (Clone: A1)	Fluidigm	3160004 B	commercially conjugated	1
162 Dy	TCRVa7.2 (Clone: 3C10)	Biolegend	351702	self-labelled	0.8
163 Dy	Anti-Human CD56 (Clone: NCAM16.2)	Fluidigm	3163007 B	commercially conjugated	0.25
164 Dy	anti-CD161 (Clone: HP-3G10)	Fluidigm	3164009 B	commercially conjugated	0.5
165 Ho	anti-human CD218a (IL-18Rα) (Clone: H44)	Biolegend	313804	self-labelled	1
166 Er	NKG2D (Clone: ON72)	Fluidigm	3166016 B	commercially conjugated	0.5
167 Er	Anti-Human CD197/CCR7 (Clone: G043H7)	Fluidigm	3167009 A	commercially conjugated	1
168 Er	anti-CD278 ICOS (Clone C398.4A)	Biolegend	313502	self-labelled	0.75
169 Tm	Anti-Human CD25 (Clone: 2A3)	Fluidigm	3169003 B	commercially conjugated	0.35
--------	Anti-humanTCR Vβ21.3-Biotin (Clone: REA894)	Miltenyi	130-114-878	commercially biotinylated	2
--------	Streptavidin-APC	Biolegend	405207	self-labelled	1
170 Er	Anti-APC (Clone: APC003)	Biolegend	408005	self-labelled	2
171 Yb	Anti-Human CD185/CXCR5 (Clone: RF8B2)	Fluidigm	3171014 B	commercially conjugated	1
172 Yb	Anti-CD27 (Clone: O323)	Biolegend	302802	self-labelled	0.8
173 Yb	anti-human CD3 (Clone: UCHT1)	Biolegend	300402	self-labelled	1
174 Yb	Anti-HLA-DR (Clone: L243)	Biolegend	307602	self-labelled	1
175 Lu	Anti-Human CD279/PD-1 (Clone: EH12.2H7)	Fluidigm	3175008 B	commercially conjugated	1
176 Yb	Anti-Human CD127/IL-7Ra (Clone: A019D5)	Fluidigm	3176004 B	commercially conjugated	0.5
195 Pt	anti-human CD45 (Clone: HI30)	Biolegend	304002	self-labelled	1
198 Pt	anti-human CD45 (Clone: HI30)	Biolegend	304002	self-labelled	1
209Bi	Anti-Human CD16 (Clone: 3G8)	Fluidigm	3209002 B	commercially conjugated	0.2

Supplementary Table 1 CyTOF T cell panel antibody list.

Metal	antibodies	SOURCE	Identifier	format	Dilution (antibody volume (ul) for 100ul staining volume)
089Y	anti-human CD45 (Clone: HI30)	Biolegend	304002	self labelled	2
106 Cd	anti-human β2-microglobulin (Clone: 2M2)	Biolegend	316302	self labelled	0.5
110 Cd	Anti-CD14 (Clone: RMO52)	Beckman Coulter	IM0643	self labelled	0.5
111 Cd	CD11b (Clone: ICRF44)	Biolegend	301306	self labelled	1
114 Cd	anti-human CD66b [Clone: 6/40c]	Biolegend	392902	self labelled	0.25
115 In	Anti-CD57 (Clone: HCD57)	Biolegend	359602	self labelled	0.5
116Cd	Anti-CD36 (Clone: 5-271)	Biolegend	336202	self labelled	0.25
141 Pr	anti-human CD84 (Clone: CD84.1.21)	Biolegend	326002	self labelled	1
142 Nd	Anti-CD19 (Clone: HIB19)	Fluidigm	3142016D	commercially conjugated	0.5
143 Nd	Anti-Human CD5 (Clone: UCHT2)	Fluidigm	3143007B	commercially conjugated	0.5
144 Nd	CD32 (Clone: FUN2)	Fluidigm	303202	self labelled	1
145 Nd	Anti-Human CD4 (Clone: RPA-T4)	Fluidigm	3145001B	commercially conjugated	0.5
147 Sm	Anti-CD11c (Clone: S-HCL-3)	Biolegend	371502	self-labelled	0.25
148 Nd	CD34 (Clone: 581)	Fluidigm	3148001B	commercially conjugated	0.4
149 Sm	Anti-CD64 (Clone: 10.1, purified)	Biolegend	305029	self labelled	0.75
150 Nd	CD69 (Clone: FN50)	Biolegend	310902	self labelled	0.75
151 Eu	anti-human CD123 (Clone: 6H6)	Biolegend	306002	self labelled	0.5
153 Eu	CX3CR1 (Clone: 2A91)	Biolegend	341602	self labelled	0.5
154 Sm	anti-human CD80 (Clone: 2D10)	Biolegend	305202	self labelled	1
155 Gd	Anti-CD45RA (Clone: HI100)	Biolegend	304102	self labelled	0.5
156 Gd	Anti-CD177 (Clone: MEM-166)	Biolegend	315802	self labelled	0.25
158 Gd	Anti-Human CD33 (Clone: WM53)	Fluidigm	3158001B	commercially conjugated	0.25
159 Tb	CD86 (Clone: FUN-1)	BD	555655	self labelled	0.25
160 Gd	Anti-Human CD39 (Clone: A1)	Fluidigm	3160004B	commercially conjugated	1
161 Dy	Anti-CD163 (Clone: GHI/61)	Biolegend	333602	self labelled	1
162 Dy	Anti-CD55 (Clone: JS11)	Biolegend	311302	self labelled	0.25
163 Dy	Anti-Human CD56 (Clone: NCAM16.2)	Fluidigm	3163007B	commercially conjugated	0.25
164 Dy	Anti-CD95 (Clone: DX2)	Biolegend	305602	self labelled	0.25
165 Ho	anti-human CD141 (Clone: M80)	Biolegend	344102	self labelled	0.25
166 Er	Anti-CD35 (Clone: E11)	Biolegend	333402	self labelled	0.25
167 Er	Anti-CD27 (Clone: L128)	Fluidigm	3167006B	commercially conjugated	0.5
168 Er	Anti-CD10 (Clone: HI10a)	Biolegend	312202	self labelled	0.25
169 Tm	Anti-Human CD25 (Clone: 2A3)	Fluidigm	3169003B	commercially conjugated	0.25
170 Er	Anti-Human CD54 (Clone: HA58)	Fluidigm	3170014B	commercially conjugated	0.25
171 Yb	CD169 (Clone: 7-239)	Fluidigm	346002	self labelled	1
172 Yb	CD71 (Clone: CY1G4)	Fluidigm	334102	self labelled	0.25
173 Yb	anti-human CD3 (Clone: UCHT1)	Biolegend	300402	self labelled	0.25
174 Yb	Anti-CD40 (Clone: HB14)	Biolegend	313002	self labelled	0.25
175 Lu	Anti-Human CD184/CXCR4 (Clone: 12G5)	Fluidigm	3175001B	commercially conjugated	1
176 Yb	Anti-CD63 (Clone: H5C6)	Biolegend	353039	self labelled	1
195 Pt	anti-human CD45 (Clone: HI30)	Biolegend	304002	self labelled	1
198 Pt	anti-human CD45 (Clone: HI30)	Biolegend	304002	self labelled	1
209Bi	Anti-Human CD16 (Clone: 3G8)	Fluidigm	3209002B	commercially conjugated	0.2

Supplementary Table 2 CyTOF Monocyte panel antibody list.

P1 T1	P1 T3	P2 T1	P2 T3	P3 T1	P3 T3	P4 T1	P4 T3	P5 T1	P5 T3
A*02:58	A*02:17:02:02	A*31:20	A*31:20	A*02:01:175	A*02:01:175	A*02:01:175	A*02:01:175	A*01:01:87	A*01:01:87
B*58:100	B*35:02:01:05	B*57:12	B*57:12	B*57:12	B*57:12	B*44:221	B*44:221	B*15:16:03	B*08:01:01:60
C*04:166:01	C*07:441:02	C*04:01:03	C*04:01:03	C*18:02:01:02	C*18:02:01:02	C*16:85	C*05:205	C*07:441:02	B*15:16:03
DPA1*01:03:17	DPA1*01:03:17	DPA1*02:01:01:29	DPA1*02:01:01:29	DPA1*01:03:17	DPA1*01:03:01:57	DPA1*01:03:17	DPA1*01:03:17	C*07:622	B
DPA1*04:02:01:02	DPA1*04:02:01:02	DPB1*13:01:01:04	DPB1*13:01:01:04	DPA1*04:02:01:02	DPA1*04:02:01:02	DPA1*02:49	DPA1*04:02:01:02	C	C*06:02:52
DPA1	DPA1	DQA1*02:01:01:03	DPB1*699:01	DPA1	DPA1	DPA1	DPA1	DPA1*02:01:02:02	C*07:441:02
DPB1*02:01:02:78	DPB1*02:01:42	DQB1*05:01:01:27	DPB1	DPB1*72:01:01:02	DPB1*23:01:01:02	DPB1*04:02:01:35	DPB1*04:02:01:35	DPA1*02:57	C
DPB1*677:01	DPB1*72:01:01:02	DRB1*07:01:01:23	DQA1*02:01:01:03	DPB1*115:01	DPB1*31:01:01:01	DPB1*677:01	DPB1*677:01	DPA1	DPA1*02:01:02:02
DPB1	DPB1		DQB1*05:01:01:27	DPB1	DPB1	DPB1	DPB1	DPB1*01:01:01:16	DPA1*02:49
DQA1*05:05:01:37	DQA1*05:05:01:37		DRB1*07:01:01:23	DQA1*01:01:02:03	DQA1*01:01:02:03	DQA1*01:01:05	DQA1*01:01:05	DQA1*01:01:01:07	DPA1
DQA1*06:01:01:03	DQA1*06:01:01:03			DQB1*05:01:01:27	DQB1*05:01:01:27	DQA1*01:25	DQA1*01:25	DQB1*05:01:01:27	DPB1*01:01:01:16
DQA1	DQA1			DRB1*01:02:01:03	DRB1*01:02:01:03	DQA1	DQA1	DRB1*01:01:01:19	DPB1*14:01:01:08
DQB1*03:01:01:50	DQB1*03:01:01:50					DQB1*05:02:01:12	DQB1*05:02:17		DPB1
DRB1*03:02:01:01	DRB1*13:11:01					DQB1*05:269	DQB1*05:159		DQA1*01:01:01:07
						DQB1	DQB1		DQB1*05:01:01:27
						DRB1*01:01:01:19	DRB1*16:01:01:01		DRB1*01:01:01:19

Supplementary Table 3 HLA phenotyping from scRNA-seq data by scHLAcount. HLA type reported to asscociated with severe MIS-C diseases (reference 3 in the main paper) were indicated in gray instead of black characters.
