# Supplementary material for: Enhanced CD95 and interleukin 18 signalling accompany T cell receptor Vβ21.3+ activation in multi-inflammatory syndrome in children
Source: Nat Commun. 2024 May 18;15:4227. doi: 10.1038/s41467-024-48699-y (PMC11102542; doi:10.1038/s41467-024-48699-y)
Supplement: Supplementary file 3 — Description of Additional Supplementary Files [file 41467_2024_48699_MOESM3_ESM.pdf]

Description of Additional Supplementary Files

Supplementary Data 1: Additional clinical and demographic information of children included in this study. (a) Clinical symptoms, SARS-Cov-2 status and treatment information for the MIS-C patients in the whole blood functional assay study. (b) Demographics of paediatric fever controls (n=16) used the whole blood functional assay study. (c) Demographics of LRTI Cohort (n=22) used in the 48-plex cytokine array study. (d) Demographics of general infection (non-covid) cohort (n=14) used in mass cytometry study.
