# Supplementary material for: Enhanced CD95 and interleukin 18 signalling accompany T cell receptor Vβ21.3+ activation in multi-inflammatory syndrome in children
Source: Nat Commun. 2024 May 18;15:4227. doi: 10.1038/s41467-024-48699-y (PMC11102542; doi:10.1038/s41467-024-48699-y)
Supplement: Supplementary file 5 — Reporting Summary [file 41467_2024_48699_MOESM5_ESM.pdf]

nature portfolio

Corresponding author(s): Nazima Pathan

Last updated by author(s): 11/03/2024

Reporting Summary

Nature Portfolio wishes to improve the reproducibility of the work that we publish. This form provides structure for consistency and transparency in reporting. For further information on Nature Portfolio policies, see our Editorial Policies and the Editorial Policy Checklist.

Statistics

For all statistical analyses, confirm that the following items are present in the figure legend, table legend, main text, or Methods section.

n/a	Confirmed	
☐	☒	The exact sample size (*n*) for each experimental group/condition, given as a discrete number and unit of measurement
☐	☒	A statement on whether measurements were taken from distinct samples or whether the same sample was measured repeatedly
☐	☒	The statistical test(s) used AND whether they are one- or two-sided<br>*Only common tests should be described solely by name; describe more complex techniques in the Methods section.*
☐	☒	A description of all covariates tested
☐	☒	A description of any assumptions or corrections, such as tests of normality and adjustment for multiple comparisons
☐	☒	A full description of the statistical parameters including central tendency (e.g. means) or other basic estimates (e.g. regression coefficient) AND variation (e.g. standard deviation) or associated estimates of uncertainty (e.g. confidence intervals)
☐	☒	For null hypothesis testing, the test statistic (e.g. *F*, *t*, *r*) with confidence intervals, effect sizes, degrees of freedom and *P* value noted<br>*Give P values as exact values whenever suitable.*
☒	☐	For Bayesian analysis, information on the choice of priors and Markov chain Monte Carlo settings
☒	☐	For hierarchical and complex designs, identification of the appropriate level for tests and full reporting of outcomes
☒	☐	Estimates of effect sizes (e.g. Cohen's *d*, Pearson's *r*), indicating how they were calculated

Our web collection on statistics for biologists contains articles on many of the points above.

Software and code

Policy information about availability of computer code

Data collection

Bio-Plex Manager 6.2 and xPONENT 4.3 were used to collect Luminex ELISA data. CyTOF Software v7.1 was used for collection of CyToF data. NovaSeq Control Software v1.7 was used to collect single cell RNA-seq data.

Data analysis

R (version 4.0.3) were used to plot the clinical test results correlation matrix with published code13, cytokine PCA map with factoextra package (version 1.0.7). OMIQ platform was used to plot the opt-tSNE map. Statistical tests of the quantitative data were performed in Graphpad Prism software (Version 9.4.0, GraphPad Software, California, USA).
For CyToF data, Flowjo (version 10.8.1) was used for analysis, as well as R package CATALYST (V1.23.4). For scRNA-seq data, these packages in python V3.9.7 were used: scanpy V1.9.1 anndata V0.8.0 umap V.5.3 numpy V1.22.3 scipy V1.8.1 pandas V1.4.3 scikit-learn V1.1.1 statsmodels V0.13.2 python-igraph V0.9.11 pynndescent V0.5.4 CellTypist V1.3.0 scHLAcount V0.2.0.
miloR V1.8.1 package was used in R V4.2.3 and other packages in R are listed below.

```
 [1] LoomExperiment_1.16.0       BiocIO_1.8.0              rhdf5_2.42.1
 [4] anndata_0.7.5.6           patchwork_1.1.2           dplyr_1.1.2
 [7] scran_1.26.2              scater_1.26.1             ggplot2_3.4.2
[10] scuttle_1.8.4             SingleCellExperiment_1.20.1 SummarizedExperiment_1.28.0
[13] Biobase_2.58.0            GenomicRanges_1.50.2      GenomeInfoDb_1.34.9
[16] IRanges_2.32.0            S4Vectors_0.36.2          BiocGenerics_0.44.0
[19] MatrixGenerics_1.10.0     matrixStats_0.63.0        miloR_1.6.0
[22] edgeR_3.40.2              limma_3.54.2

loaded via a namespace (and not attached):
```

```
[1] bitops_1.0-7            RColorBrewer_1.1-3        rprojroot_2.0.3
[4] tools_4.2.3             utf8_1.2.3                R6_2.5.1
[7] irlba_2.3.5.1           HDF5Array_1.26.0          vipor_0.4.5
[10] DBI_1.1.3              colorspace_2.1-0          rhdf5filters_1.10.1
[13] withr_2.5.0            tidyselect_1.2.0          gridExtra_2.3
[16] compiler_4.2.3         cli_3.6.1                 BiocNeighbors_1.16.0
[19] DelayedArray_0.23.2    scales_1.2.1              stringr_1.5.0
[22] digest_0.6.31          rmarkdown_2.21            XVector_0.38.0
[25] pkgconfig_2.0.3        htmltools_0.5.5           sparseMatrixStats_1.10.0
[28] fastmap_1.1.1          rlang_1.1.0               rstudioapi_0.14
[31] DelayedMatrixStats_1.20.0 farver_2.1.1           generics_0.1.3
[34] jsonlite_1.8.4         BiocParallel_1.32.6       gtools_3.9.4
[37] RCurl_1.98-1.12        magrittr_2.0.3            BiocSingular_1.14.0
[40] GenomeInfoDbData_1.2.9 Matrix_1.5-3              Rhdf5lib_1.20.0
[43] Rcpp_1.0.10            ggbeeswarm_0.7.2          munsell_0.5.0
[46] fansi_1.0.4            reticulate_1.28           viridis_0.6.3
[49] lifecycle_1.0.3        stringi_1.7.12            yaml_2.3.7
[52] ggraph_2.1.0           MASS_7.3-60               zlibbioc_1.44.0
[55] grid_4.2.3             dqrng_0.3.0               parallel_4.2.3
[58] ggrepel_0.9.3          lattice_0.21-8            graphlayouts_1.0.0
[61] cowplot_1.1.1          beachmat_2.14.2           locfit_1.5-9.7
[64] metapod_1.6.0          knitr_1.42                pillar_1.9.0
[67] igraph_1.4.3           codetools_0.2-19          ScaledMatrix_1.6.0
[70] glue_1.6.2             evaluate_0.21             png_0.1-8
[73] vctrs_0.6.2            tweenr_2.0.2              gtable_0.3.3
[76] purrr_1.0.1            polyclip_1.10-4           tidyr_1.3.0
[79] assertthat_0.2.1       xfun_0.38                 ggforce_0.4.1
[82] rsvd_1.0.5             tidygraph_1.2.3           viridisLite_0.4.2
[85] tibble_3.2.1           beeswarm_0.4.0            cluster_2.1.4
[88] statmod_1.5.0          bluster_1.8.0             here_1.0.1
```

For manuscripts utilizing custom algorithms or software that are central to the research but not yet described in published literature, software must be made available to editors and reviewers. We strongly encourage code deposition in a community repository (e.g. GitHub). See the Nature Portfolio guidelines for submitting code & software for further information.

Data

Policy information about availability of data

All manuscripts must include a data availability statement. This statement should provide the following information, where applicable:

- Accession codes, unique identifiers, or web links for publicly available datasets
- A description of any restrictions on data availability
- For clinical datasets or third party data, please ensure that the statement adheres to our policy

The scRNA-Seq data generated in this study have been deposited in the Zenodo database under accession code 7997382, https://zenodo.org/records/7997382. The CyToF data from the T cell panel study have been deposited in the FlowRepository database under access code FR-FCM-Z6F8, http://flowrepository.org/id/FR-FCM-Z6F8.

Human research participants

Policy information about studies involving human research participants and Sex and Gender in Research.

Reporting on sex and gender	In the study, both male and female participants were recruited. We have a summary of the gender information in Figure 1. No gender specific effect was analyzed due to limited sample size.
Population characteristics	We provided population characteristics in Figure 1, including age, weight and ethnicity.
Recruitment	Patients were recruited from 3 paediatric intensive care units in UK. Informed consent was obtained (from parents or guardians of children <16years of age) by trained health professionals. There was no selection bias when recruiting MIS-C patients.
Ethics oversight	The study enrolled children consented to the TrICICL study (South of Birmingham Research Ethics Committee, REC reference 17/WM/0453), or the DIAMONDS-Search study (approved by London-Dulwich Research Ethics Committee, REC reference 20/HRA/1714) or the RASCALS study (approved by Yorkshire and the Humber-Bradford Leeds Research Ethics Committee, REC reference 20/YH/0089).

Note that full information on the approval of the study protocol must also be provided in the manuscript.

Field-specific reporting

Please select the one below that is the best fit for your research. If you are not sure, read the appropriate sections before making your selection.

☒ Life sciences ☐ Behavioural & social sciences ☐ Ecological, evolutionary & environmental sciences

For a reference copy of the document with all sections, see nature.com/documents/nr-reporting-summary-flat.pdf

Life sciences study design

All studies must disclose on these points even when the disclosure is negative.

Sample size	No sample size calculation were performed. We aim to get more than 10 samples per group. This number is selected by checking literature in MIS-C studies.
Data exclusions	No data were excluded.
Replication	Replication is not possible for the study. Human samples of MIS-C blood were limited and the experiment methods were costly.
Randomization	Randomization is not relevant to the study. We are trying to explore disease mechanism, while randomization is usually used for testing treatment effect. However, in the cytof experiment, we did randomize samples from different groups in the same tube.
Blinding	Blinding was possible at all stages as we are a small research team. However, in cytof experiment, the machine operator was blind to sample groupings.

Reporting for specific materials, systems and methods

We require information from authors about some types of materials, experimental systems and methods used in many studies. Here, indicate whether each material, system or method listed is relevant to your study. If you are not sure if a list item applies to your research, read the appropriate section before selecting a response.

Materials & experimental systems

n/a	Involved in the study
☐	☒ Antibodies
☒	☐ Eukaryotic cell lines
☒	☐ Palaeontology and archaeology
☒	☐ Animals and other organisms
☐	☒ Clinical data
☒	☐ Dual use research of concern

Methods

n/a	Involved in the study
☒	☐ ChIP-seq
☐	☒ Flow cytometry
☒	☐ MRI-based neuroimaging

Antibodies

Antibodies used

T cell panel:
Metal antibodies Source Identifier format Dilution (antibody volume (ul) for 100ul staining volume)
089Y anti-human CD45 (Clone: HI30) Biolegend 304002 self labelled 2
106 Cd anti-human β2-microglobulin (Clone: 2M2) Biolegend 316302 self-labelled 0.5
110 Cd Anti-CD14 (Clone: RMO52) Beckman Coulter IM0643 self labelled 0.5
111 Cd Anti-CD38 (Clone: HIT2) Biolegend 303502 self-labelled 0.75
114 Cd Anti-CD8 (Clone: RPA-T8) Biolegend 301002 self labelled 0.4
115 In Anti-CD57 (Clone: HCD57) Biolegend 359602 self-labelled 0.5
141 Pr CCR6 (Clone: G034E3) Biolegend 353402 self-labelled 0.7
142 Nd Anti-CD19 (Clone: HIB19) Fluidigm 3142016D commercially conjugated 0.5
143 Nd Anti-Human CD5 (Clone: UCHT2) Fluidigm 3143007B commercially conjugated 0.5
145 Nd Anti-Human CD4 (Clone: RPA-T4) Fluidigm 3145001B commercially conjugated 0.5
146 Nd anti-TIGIT (Clone: A15153G) Biolgend 372702 self-labelled 1
147 Sm anti-human CD366 (Tim-3) (Clone: F38-2E2) Biolegend 345002 self-labelled 1
148 Nd Anti-CD95 (Clone: DX2, purified) Biolegend 305602 self-labelled 1
149 Sm CCR4 (Clone: L291H4) Biolegend 359402 self-labelled 0.5
150 Nd Anti-Human CD223/LAG-3 (Clone: 11C3C65) Fluidigm 3150030B commercially conjugated 0.5
151 Eu TCRVa24 (Clone: 6B11) Biolegend 342902 self-labelled 1
152 Sm Anti-Human TCRgd (Clone: 11F2) Fluidigm 3152008B commercially conjugated 1
153 Eu anti-CX3CR1 (Clone: 2A91) Biolegend 341602 self-labelled 1

154 Sm anti-human IgD (Clone: IA6-2) Biolegend 348202 self-labelled 0.25
155 Gd anti-CD45RA (Clone: HI100) Fluidigm 3155011B commercially conjugated 0.5
156 Gd anti-CXCR3 (Clone: G025H7) Fluidigm 3156004B commercially conjugated 0.7
159 Tb CD28 (Clone:CD28.2) Biolegend 302902 self-labelled 0.75
160 Gd Anti-Human CD39 (Clone: A1) Fluidigm 3160004B commercially conjugated 1
162 Dy TCRVa7.2 (Clone: 3C10) Biolegend 351702 self-labelled 0.8
163 Dy Anti-Human CD56 (Clone: NCAM16.2) Fluidigm 3163007B commercially conjugated 0.25
164 Dy anti-CD161 (Clone: HP-3G10) Fluidigm 3164009B commercially conjugated 0.5
165 Ho anti-human CD218a (IL-18Rα) (Clone: H44) Biolegend 313804 self-labelled 1
166 Er NKG2D (Clone: ON72) Fluidigm 3166016B commercially conjugated 0.5
167 Er Anti-Human CD197/CCR7 (Clone: G043H7) Fluidigm 3167009A commercially conjugated 1
168 Er anti-CD278 ICOS (Clone C398.4A) Biolegend 313502 self-labelled 0.75
169 Tm Anti-Human CD25 (Clone: 2A3) Fluidigm 3169003B commercially conjugated 0.35
-------- Anti-humanTCR Vβ21.3-Biotin (Clone: REA894) Miltenyi 130-114-878 commercially biotinylated 2
-------- Streptavidin-APC Biolegend 405207 self-labelled 1
170 Er Anti-APC (Clone: APC003) Biolegend 408005 self-labelled 2
171 Yb Anti-Human CD185/CXCR5 (Clone: RF8B2) Fluidigm 3171014B commercially conjugated 1
172 Yb Anti-CD27 (Clone: O323) Biolegend 302802 self-labelled 0.8
173 Yb anti-human CD3 (Clone: UCHT1) Biolegend 300402 self-labelled 1
174 Yb Anti-HLA-DR (Clone: L243) Biolegend 307602 self-labelled 1
175 Lu Anti-Human CD279/PD-1 (Clone: EH12.2H7) Fluidigm 3175008B commercially conjugated 1
176 Yb Anti-Human CD127/IL-7Ra (Clone: A019D5) Fluidigm 3176004B commercially conjugated 0.5
195 Pt anti-human CD45 (Clone: HI30) Biolegend 304002 self-labelled 1
198 Pt anti-human CD45 (Clone: HI30) Biolegend 304002 self-labelled 1
209Bi Anti-Human CD16 (Clone: 3G8) Fluidigm 3209002B commercially conjugated 0.2

Monocyte panel:
Metal antibodies SOURCE Identifier format Dilution (antibody volume (ul) for 100ul staining volume)
089Y anti-human CD45 (Clone: HI30) Biolegend 304002 self labelled 2
106 Cd anti-human β2-microglobulin (Clone: 2M2) Biolegend 316302 self labelled 0.5
110 Cd Anti-CD14 (Clone: RMO52) Beckman Coulter IM0643 self labelled 0.5
111 Cd CD11b (Clone: ICRF44) Biolegend 301306 self labelled 1
114 Cd anti-human CD66b [Clone: 6/40c] Biolegend 392902 self labelled 0.25
115 In Anti-CD57 (Clone: HCD57) Biolegend 359602 self labelled 0.5
116Cd Anti-CD36 (Clone: 5-271) Biolegend 336202 self labelled 0.25
141 Pr anti-human CD84 (Clone: CD84.1.21) Biolegend 326002 self labelled 1
142 Nd Anti-CD19 (Clone: HIB19) Fluidigm 3142016D commercially conjugated 0.5
143 Nd Anti-Human CD5 (Clone: UCHT2) Fluidigm 3143007B commercially conjugated 0.5
144 Nd CD32 (Clone: FUN2) Fluidigm 303202 self labelled 1
145 Nd Anti-Human CD4 (Clone: RPA-T4) Fluidigm 3145001B commercially conjugated 0.5
147 Sm Anti-CD11c (Clone: S-HCL-3) Biolegend 371502 self-labelled 0.25
148 Nd CD34 (Clone: 581) Fluidigm 3148001B commercially conjugated 0.4
149 Sm Anti-CD64 (Clone: 10.1, purified) Biolegend 305029 self labelled 0.75
150 Nd CD69 (Clone: FN50) Biolegend 310902 self labelled 0.75
151 Eu anti-human CD123 (Clone: 6H6) Biolegend 306002 self labelled 0.5
153 Eu CX3CR1 (Clone: 2A91) Biolegend 341602 self labelled 0.5
154 Sm anti-human CD80 (Clone: 2D10) Biolegend 305202 self labelled 1
155 Gd Anti-CD45RA (Clone: HI100) Biolegend 304102 self labelled 0.5
156 Gd Anti-CD177 (Clone: MEM-166) Biolegend 315802 self labelled 0.25
158 Gd Anti-Human CD33 (Clone: WM53) Fluidigm 3158001B commercially conjugated 0.25
159 Tb CD86 (Clone: FUN-1) BD 555655 self labelled 0.25
160 Gd Anti-Human CD39 (Clone: A1) Fluidigm 3160004B commercially conjugated 1
161 Dy Anti-CD163 (Clone: GHI/61) Biolegend 333602 self labelled 1
162 Dy Anti-CD55 (Clone: JS11) Biolegend 311302 self labelled 0.25
163 Dy Anti-Human CD56 (Clone: NCAM16.2) Fluidigm 3163007B commercially conjugated 0.25
164 Dy Anti-CD95 (Clone: DX2) Biolegend 305602 self labelled 0.25
165 Ho anti-human CD141 (Clone: M80) Biolegend 344102 self labelled 0.25
166 Er Anti-CD35 (Clone: E11) Biolegend 333402 self labelled 0.25
167 Er Anti-CD27 (Clone: L128) Fluidigm 3167006B commercially conjugated 0.5
168 Er Anti-CD10 (Clone: HI10a) Biolegend 312202 self labelled 0.25
169 Tm Anti-Human CD25 (Clone: 2A3) Fluidigm 3169003B commercially conjugated 0.25
170 Er Anti-Human CD54 (Clone: HA58) Fluidigm 3170014B commercially conjugated 0.25
171 Yb CD169 (Clone: 7-239) Fluidigm 346002 self labelled 1
172 Yb CD71 (Clone: CY1G4) Fluidigm 334102 self labelled 0.25
173 Yb anti-human CD3 (Clone: UCHT1) Biolegend 300402 self labelled 0.25
174 Yb Anti-CD40 (Clone: HB14) Biolegend 313002 self labelled 0.25
175 Lu Anti-Human CD184/CXCR4 (Clone: 12G5) Fluidigm 3175001B commercially conjugated 1
176 Yb Anti-CD63 (Clone: H5C6) Biolegend 353039 self labelled 1

195 Pt anti-human CD45 (Clone: HI30) Biolegend 304002 self labelled 1
198 Pt anti-human CD45 (Clone: HI30) Biolegend 304002 self labelled 1
209Bi Anti-Human CD16 (Clone: 3G8) Fluidigm 3209002B commercially conjugated 0.2

Validation	We validated our TCR Vbeta21.3 antibody staining in healthy blood samples. For the antibodies used in the cytof experiments, we validated them using in vitro stimulated blood cone cells.

Clinical data

Policy information about clinical studies
All manuscripts should comply with the ICMJEguidelines for publication of clinical research and a completedCONSORT checklist must be included with all submissions.

Clinical trial registration	*Provide the trial registration number from ClinicalTrials.gov or an equivalent agency.*
Study protocol	*Note where the full trial protocol can be accessed OR if not available, explain why.*
Data collection	*Describe the settings and locales of data collection, noting the time periods of recruitment and data collection.*
Outcomes	*Describe how you pre-defined primary and secondary outcome measures and how you assessed these measures.*

Flow Cytometry

Plots

Confirm that:

- [x] The axis labels state the marker and fluorochrome used (e.g. CD4-FITC).
- [x] The axis scales are clearly visible. Include numbers along axes only for bottom left plot of group (a 'group' is an analysis of identical markers).
- [x] All plots are contour plots with outliers or pseudocolor plots.
- [x] A numerical value for number of cells or percentage (with statistics) is provided.

Methodology

Sample preparation	To collect PBMCs, whole blood was mixed with DPBS at 1:1 ratio before being laid on top of a Leucosep tube containing Ficoll Paque Premium. The tube was centrifuged for 10min with brake off at 1000g at room temperature. The PBMC pellet was washed twice before being resuspended in FBS solution containing 10% DMSO for freezing -150 degree freezer. Freshly thawed PBMC samples were used for the staining.
Instrument	Helios CyToF machine, made by Standard BioTools.
Software	CyTOF Software v7.1 was used for collection of CyToF data. www.OMIQ.ai was used for tSNE plotting. Flowjo (version 10.8.1) was used for analysis, as well as R package CATALYST (V1.23.4)
Cell population abundance	No cell sorting was perfomed.
Gating strategy	Gating strategy was illustrated with supplementary figures 2 and 3.

- [x] Tick this box to confirm that a figure exemplifying the gating strategy is provided in the Supplementary Information.
